# Neutrophil swarm control: what goes up must come down

**DOI:** 10.1038/s41392-021-00836-5

**Published:** 2021-12-06

**Authors:** Stefan Uderhardt, Jasmin Knopf, Martin Herrmann

**Affiliations:** 1 Department of Internal Medicine 3—Rheumatology and Immunology, Friedrich‐Alexander‐University Erlangen‐Nürnberg (FAU) and Universitätsklinikum Erlangen, Erlangen, Germany; 2 Deutsches Zentrum für Immuntherapie (DZI), Friedrich‐Alexander‐University Erlangen‐Nürnberg (FAU) and Universitätsklinikum Erlangen, Erlangen, Germany; 3 Exploratory Research Unit, Optical Imaging Centre Erlangen, Friedrich‐Alexander‐University Erlangen‐Nürnberg (FAU), Erlangen, Germany

**Keywords:** Innate immune cells, Inflammation

All biological systems rely on regulatory networks of positive and negative feedback that enable targeted and rapid adaptations, while negative autosignals limit overstimulation and enable self-shutdown. A recent publication in *Science*^1^ demonstrates a shutdown mechanism in neutrophils that limits their aggregation dynamics while enhancing bacterial killing.^1^

**Fig. 1:**
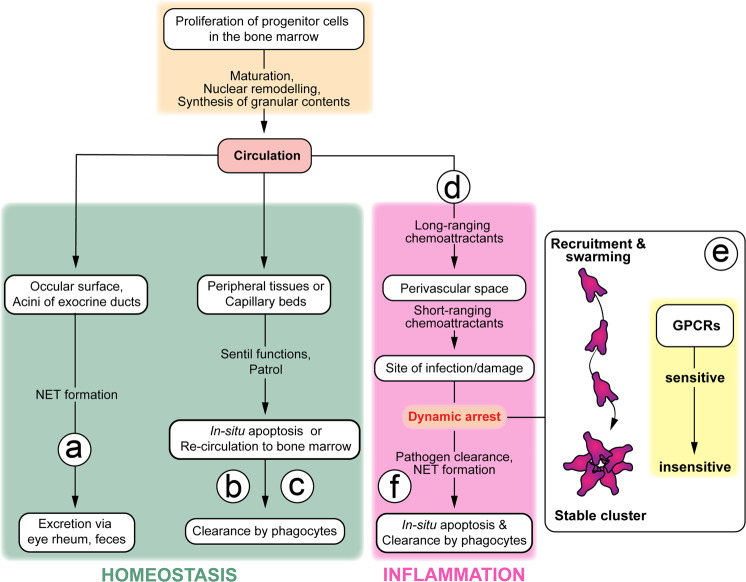
Neutrophil fates under homeostatic or inflammatory conditions. Neutrophils arise from precursor cells in the bone marrow and are released into the bloodstream, from where they set out to patrol the internal tissues as well as the ocular surfaces and exocrine ducts. Under homeostatic conditions (green box), neutrophils undergo different fates: (a) NET formation and excretion, (b) in situ apoptosis or (c) re-circulation to the bone marrow and clearance. Under inflammatory conditions (red box), neutrophils are attracted to the site of infection or damage (d) first through a sequential combination of long- and short-ranging chemoattractants. The latter mediates the coordinated extravascular aggregation dynamics known as swarming behavior. Kienle et al.^1^ now show that preprogrammed desensitization of the very G-protein-coupled receptors (GPCRs) that drive swarming is an intrinsic feedback control that leads to dynamic arrest (e). This arrest ensures stable cluster formation and is critical for their antibacterial effector functions. Successful elimination of the invading pathogen is then followed by apoptosis in situ and elimination by recruited phagocytes (f), which is the trigger for the active resolution of inflammation

The inflammatory cascade is a highly conserved process that allows for the orderly activation and deactivation of immune cells in response to challenges from within or the outside. One of its most numerous and powerful effectors are neutrophil granulocytes. Neutrophils exert their inflammatory functions as functional collective through a highly dynamic behavior called swarming. Swarming dynamics are a universal feature of neutrophil activation and their early recruitment in response to a variety of inflammatory stimuli and perturbations, including sterile tissue damage. The molecular mechanisms of swarming enable the targeted and incredibly rapid recruitment of inflammatory effectors to sites of potential barrier breach or pathogen invasion. In particular, the initial self-amplifying mechanisms are extremely effective. In situ activation of an individual sentinel is sufficient to initiate a feedforward circuit based on the secretion of the chemokines LTB4 and CCL2. This leads to the recruitment and targeted attraction of hundreds of activated neutrophils from circulation—like the feeding frenzy of piranhas. However, the activity of neutrophils comes at a price. Neutrophils carry powerful molecular weapons that cannot distinguish between invading bacteria and the body’s own cells. While swarming is essential for fighting pathogens, it inevitably also inflicts collateral damage on healthy tissue, which has been implicated in the pathogenesis of various inflammatory and degenerative diseases.^2^ So, while the initial steps and molecular pathways become increasingly clear, observing a swarm event in full swing almost immediately raises the question: when and how does it stop? Such an important and potentially harmful process must be regulated at multiple levels. Both intrinsic and extrinsic cellular mechanisms operate in parallel, depending on the actual in vivo context. The different fates of neutrophils are shown in Fig. 1.

For instance, monocytes have been implicated in controlling the population dynamics of swarming neutrophils. In vivo, the cessation of swarm progression and further recruitment of neutrophils is associated with secondary shielding of the focus by recruited anti-inflammatory monocytes.^3^ These cells were shown to closely follow the first wave of infiltrating neutrophils. Here, degradation of neutrophil chemoattractants or even their molecular conversion to neutrophil-deactivating mediators could limit swarm growth and keep the inflammatory process in check. In addition, upon activation, neutrophils undergo neutrophil extracellular trap (NET) formation—a particular form of cell death associated with the release of neutrophil-derived chromatin associated with various enzymes and proteins. NETs have been shown to be enriched in proteases that actively degrade proinflammatory mediators as well as various chemokines.^4,5^ Although NETs are difficult to detect in the core of an active swarm cluster, we speculate that they would represent a powerful negative feedback mechanism that self-limits neutrophil activation by providing a temporary (pop-up) chemokine-degrading scaffold.

However, because it is so critical to effectively regulate the neutrophil activity, each cell has its own control mechanisms in the form of molecular circuit breakers. For example, it has long been known that circulating neutrophils are programmed for constitutive cell death. Their inherently limited lifespan can be considered a safety measure to limit their destructive potential, which is why the temporal extent of a neutrophil-driven inflammatory activity is primarily regulated by sustained replenishment from the bone marrow. Kienle et al.^1^ now present a novel intrinsic feedback control at the level of regulation of individual cell dynamics. Their finding is based on the observation that once neutrophils have swarmed and cluster, they exhibit dynamic arrest, whereas cells at the periphery exhibit intermittent exploratory behavior. They show that this arrest is mediated by active desensitization of the LTB4/CCL2-driven signaling pathways in activated neutrophils. GRK2-mediated phosphorylation of the respective surface receptors leads to reduced receptivity of clustering neutrophils even and especially at increasing chemokine concentrations—a shift from initial positive (swarming) to subsequent negative temporal sensing (swarm aggregation). While GRK2 signaling has previously been implicated in chemotaxis of immune cells, in extravascular neutrophils GRK2-mediated arrest limits dynamic aggregation and thus the progression of cluster formation, which may be important in limiting collateral tissue damage and pathological inflammation in response to sterile disruptions.^3^ However, in the case of bacterial infection, this arrest is of even greater functional significance. The spatiotemporal dynamics of neutrophils in response to infections and sterile tissue damage are strikingly similar and rely on the same positive feedback loops. While the direct benefit of swarming in sterile lesions remains speculative, their role in the containment and control of bacterial infections is evident. In a mouse model of lymph node infection, the authors found that although non-arresting neutrophils exhibited increased aggregation and exploratory dynamics throughout the tissue, bacterial clearance was diminished. They conclude that dynamic arrest of neutrophils is a prerequisite for the formation of stable clusters and thus for the effective killing of bacteria. Without GRK2-mediated arrest, neutrophils do not commit to cluster stability, rendering cluster functionality less effective. Instead, the cells tend to engage in exploratory behavior or constantly migrate between different clusters. However, at the functional level, exploration alone is not sufficient to eliminate the threat. Instead, swarm-driven formation and maintenance of functional clusters are essential for killing bacteria. Turning off their responsiveness to the chemokine that actually attracted them there, exactly where its concentration is highest, ensures that they are present where they are needed most, at the center of the swarm.

Overall, mechanisms for (self-)limiting neutrophil dynamics are crucial for the prevention of collateral tissue damages and chronic inflammatory diseases. In addition, however, they also enable a more targeted use of their powerful effector functions by actively directing their focus to the task at hand. In summary, neutrophil activation and deactivation are dynamic processes that are regulated at multiple levels, both cell-intrinsic and -extrinsic. All mechanisms must be closely coordinated and effectively interact to enable the delicate balance between self-destruction and the main purpose of inflammation—protection from the outside world.
